# Novel L-lysine α-oxidase from marine streptomyces: production, optimization and potent antibacterial activity against drug-resistant pathogens

**DOI:** 10.1186/s12934-026-02992-1

**Published:** 2026-04-13

**Authors:** Mohamed Abdelraof, Amany A. Hassabo, Doaa Darwich, Mohamed M. Abdel-Monsef

**Affiliations:** 1 Microbial Chemistry Department, Biotechnology Research Institute, National Research Centre, Giza, 12622 Dokki Egypt; 2 Molecular Biology Department, National Research Centre, El-Tahrir St., Dokki, Giza, Egypt; 3 Proteome Research Laboratory, Central Laboratories Network and Centers of Excellence, National Research Centre, El-Tahrir St., Dokki, Giza, Egypt

**Keywords:** L-lysine α-oxidase, *Streptomyces griseobrunneus*, Optimization, Purification, Antibacterial

## Abstract

This study reported the optimization, purification and characterization of a novel L-lysine α-oxidase (LLO) from marine-derived *Streptomyces griseobrunneus* strain S15. Among the twenty-two Streptomyces isolates, S15 showed the highest production of LLO (8 U/mg) after 96 h of cultivation. Molecular identification via 16S rRNA sequencing confirmed its phylogenetic relationship to *S. griseobrunneus* (GenBank PQ416578). Optimization of LLO production using response surface methodology enhanced the enzyme production by about five-fold compared to the control. Purification of LLO resulted in a yield of 49.99% and 2.356-fold purification through a single-step Sephacryl S-300 column. The purified homodimeric enzyme (115 kDa native, 58 kDa subunit) exhibited optimal activity at pH 6.4 and 50 °C, with exceptional thermal stability at physiological temperatures. According to kinetic studies, the enzyme has a high substrate affinity (***K***_***m***_ 0.050 mM for L-lysine) and a broad specificity for basic and hydrophobic amino acids. Moreover, the enzyme showed potent antibacterial effects against multidrug-resistant pathogens (MIC 1.99–5.33 U/mL), demonstrated comparable or enhanced antibacterial activity relative to tested antibiotics. The unique combination of catalytic efficiency, stability, and antimicrobial activity makes this LLO a promising candidate for therapeutic and industrial applications.

## Introduction

Human health still faces major risks in recent years, such as infections, cancer, and more dangerously antibiotic-resistant bacteria [1, 2]. The development of novel, effective natural therapeutics is of the highest priority. Over the past 40 years, extensive research has been conducted on cancer therapy that utilizes the cleavage of specific amino acids from blood serum levels by enzymes [3–5].

Amino acid degrading enzymes like L-Amino acid oxidases (LAAOs) have shown prospective applications in biomedicine and in the treatment of several diseases including microbial infections, autoimmune diseases and cancer [6, 7]. LAAOs are flavoenzymes, which oxidatively deaminate L-amino acids to the corresponding α-keto acids, producing ammonia and hydrogen peroxide. They have been widely expressed from both prokaryotic and eukaryotic cells in many species [3, 8, 9]. A promising antimicrobial and anticancer LAAO was isolated from *Trichoderma harzianum* that demonstrated remarkable catalytic specificity for L-lysine [5]. These LAAOs are called according to the specificity of their substrates like lysine oxidase which is the subject of the current study.

L-lysine α-oxidase (LLO) is one of L-amino acid oxidases which catalysis deamination of L-lysine into α-keto-ε-aminocaproate, H2O2 and NH_3_. The enhanced therapeutic value of LLO may be due to their significant antibacterial, antifungal, antiviral, and anti-HIV properties. [2, 5]. LLO has been comprehensively characterized from bacteria and various *Trichoderma* sp., including *Trichoderma aureoviride*, and *Trichoderma harzianum*, that appear to be more frequent sources of the enzyme [10–12] [13, 14]. The first LLO preparation was obtained from *Trichoderma viride* Y244 with a yield of 8% [15]. LLO was obtained from *Pseudomonas* sp. in the presence of lysine as a nitrogen source and exhibited the optimal activity at pH 7.0 [16]. LLOs purified from *Trichoderma viride* and *Pseudomonas* sp. are enzymatically active as homodimers with molecular mass of 100–120 kDa [10, 15, 16]. In addition, LLO was purified from *Pseudomonas aeruginosa* and demonstrated potent cytotoxicity against colorectal cancer cells [17]. Isobe and Nagasawa (2007) [10] characterized LLO from cell free extract of *Rhodococcus* sp which has a molecular mass of 100 kDa. Sanchez-Amat et al. (2010) [18] isolated the marine bacterium *Marinomonas mediterranea*, with unique traits that make it powerful tool for discovering a novel LLO. However, there are currently no published reports on the purification of LLO from *Streptomyces* sp. Since every organism has different requirements, designing suitable cultural conditions is crucial for enhancing economically bioprocesses. Response surface methodology (RSM) is a modeling method that determines the relationship between the independent variables and the responses. Additionally, regression analysis is used to examine the impact of the variables separately or in combination [19].

There are several biological effects of LLO that have been documented, including antibacterial, antiviral, anti-protozoa, and anticancer properties [11, 18]. In this respect, LLO obtained from *Trichoderma aureoviride* directly leads to death of the pathogen by triggering apoptosis [2]. The potential of LLO in developing treatments for antibiotic-resistant bacteria is demonstrated by its antagonistic effects on both Gram-positive and Gram-negative bacteria, as well as phytopathogenic fungi, leading to cell death [2, 5, 21]. The antibacterial activity of LLO may be achieved through two pathways: accumulation of H₂O₂ from catalytic reactions to damage bacterial cell structures, and inhibition of bacterial metabolism by depleting L-lysine essential for growth [22, 23]. The direct cytotoxicity and generation of reactive oxygen species are the causes of DNA damage and cell death [6]. Previous studies have demonstrated that catalases typically suppress the bactericidal activity of LLOs demonstrating that the hydrogen peroxide produced by LLOs is responsible for their antibacterial effect [6, 20]. Moreover, it has been demonstrated that LLO has significant potential as an anticancer agent, as it effectively reduces L-lysine levels both in vitro and in vivo [3, 18]. It has been observed that leukemic cell proliferation is inhibited when L-lysine is removed from the culture media, indicating that tumor cell growth requires a high concentration of L-lysine [3, 11]. Several investigations showed that LLO exhibited concentration-dependent cytotoxicity, which resulted in either necrosis or apoptosis, suggesting that it could be a promising anticancer drug [6, 22]. In this concern, *Trichoderma aureoviride* LLO demonstrated a cytotoxic effect on various cancer cells, such as erythromyeloblastic leukemia, colon, and breast cancer, in a process that is dependent on concentration and time [2].

Isolation from marine habitat could provide newly producers of LLO, which may relate to new enzymatic characterization. Considering the therapeutic potential of LLO as well as the fact that *Streptomycetes* have not yet been investigated for production of LLO, therefore, this study offers insight for the first time into optimization and purification of LLO from *Streptomyces* isolates as a promising therapeutic agent. Statistical experimental designs were used to optimize the significant variables that affect the production of the enzyme. Purification and characterization of LLO from potent *Streptomyces* producer was carried out. Moreover, we evaluate the antimicrobial potential of pure enzyme against clinically important bacterial pathogens.

## Materials and methods

### Materials

L-Lysine, o-phenylenediamine (OPD), horseradish peroxidase (HRP), Sephacryl S-300, bovine serum albumin (BSA), and kits of gel filtration molecular weight marker were from Sigma Co. Marker proteins of SDS molecular weight were purchased from Pharmacia Co. All other chemicals of analytical grade.

### Isolation of marine *Streptomyces* sp. and inoculum preparation

Marine *Streptomyces* isolates, used in our study, were isolated from marine environment (sea water and sand beach) of the Red Sea, Hurghada Egypt (27◦13′ N 33◦56′E). Ten grams of each sand and sea water sample were suspended in 90 mL sterile seawater and serially diluted up to 10⁻⁶. Aliquots (100 µL) from appropriate dilutions were spread onto starch nitrate agar supplemented with 3% (w/v) sea salt and cycloheximide (50 µg/mL) to inhibit fungal growth. Plates were incubated at 30 °C for 7–14 days. Colonies showing typical *Streptomyces* morphology (powdery appearance, tough leathery texture, and aerial mycelium formation) were sub-cultured repeatedly to obtain pure isolates, which were maintained on starch nitrate agar slants at 4 °C for further analysis [25]. For inoculum preparation, *Streptomyces* suspension was obtained by scraping the surface of slants in sterile distilled water (10 ml) to made heavy spore concentration of about 10^6^–10^7^ Colony Forming Unit (CFU).

#### Qualitative screening of the LLO-*Streptomyces* producers

Screening of the LLO production by the *Streptomyces* isolates was applied using the Prussian blue agar test with some modification [24]**.** *Streptomyces* isolates (10^6^–10^7^ CFU/ml) were inoculated in 250 ml Erlenmeyer flasks containing enzyme production medium composed of 2% Starch, 0.5% peptone, 0.3% yeast extract, 3% sea salt, and supplemented with 0.1% L-lysine as inducer [2]. The cultures were incubated with shaking (180 rpm) at 30 °C. After 96 h, the culture supernatant was separated by centrifugation at 8000 rpm for 10 min at 4 °C and represented as a crud enzyme. The detection of LLO was carried out using a qualitative method [26]. Briefly, after cultivation of *Streptomyces* isolates on the lysine-supplemented medium, aliquots (50 μl) of the resulting supernatant for each isolate were transferred into circle wells (6 mm) of Prussian blue agar and then the plates were left at room temperature for 30 min. The color change was considered as a positive result, and the size of the blue hole was measured for each isolate. Potent LLO-producing *Streptomyces* isolates were selected based on the color change diameter (mm), and further detection of LLO was carried out by a quantitative assay method.

#### Quantitative LLO assay

To determine lysine oxidase (LLO) enzymatic activity, a colorimetric microplate-based assay was performed in duplicate, adapted from established methodologies [27]. Each 100 µL assay contained 20 µL of enzyme sample and 80 µL of a reaction solution. This solution was composed of 50 mM Tris–HCl buffer (pH 8.0), 5 mM L-lysine as the primary substrate, 0.81 U/mL horseradish peroxidase (HRP), and 2 mM o-phenylenediamine (OPD) as the chromogenic agent. Following a 60-min incubation period at 37 °C, the enzymatic reaction was terminated by adding 20 µL of 2 M sulfuric acid (H₂SO₄). The amount of hydrogen peroxide (H₂O₂) generated, which correlates directly with LLO activity, was determined by measuring the optical density at 490 nm using a BioTek ELx800 microplate reader (Agilent Technologies, USA). Enzyme activity was interpolated from a standard curve prepared with known concentrations of H₂O₂. One unit (U) of LLO activity was defined as the quantity of enzyme that catalyzes the formation of 1 µM of H₂O₂ per minute. The results are expressed as specific activity, calculated in units per milligram of total protein (U/mg).

#### Molecular identification of the potent-producer

The best *Streptomyces* producer demonstrated the maximum enzyme productivity was selected to the genetic identification via sequencing of 16 s rRNA. In this way, molecular characterization of the targeted isolate included total genomic DNA extraction, PCR amplification, purification, and sequencing were performed using a protocol of Macrogen Company (Seoul, South Korea; https://www.macrogen.com). Amplification of genetic material was taken place using the universal forward primer 8F (5′-CAG GCC TAA CAC ATG CAA GTC-3′) and reverse primer 1492R (5′-GGG CGG GGT GTACAA GGC-3′). The PCR mixture was applied in a volume of 50 μl, containing 22 μl of MQ, 25 μl of DreamTaq Green DNA Polymerase (Thermo Fisher Scientific, USA), 1 μl of each forward and reverse primer (10 μmol/L), and 1 μl of template. The PCR amplification conditions were 4 min of preheating at 95 °C, 30 s denaturation at 95 °C, 45 s of primer annealing at 50 °C, 1 min extension step at 72 °C, and post cycling extension of 10 min at 72 °C for 35 cycles [28]. The reactions were carried out in a thermal cycler (Applied Biosystem Thermal Cycler, USA). Followed by, aligned of the resulting sequences and compared with the sequences deposited in GenBank (http://blast.ncbi.nlm.nih.gov/Blast.cgi) was implemented using the Basic Local Alignment Search Tool (BLAST). Bioinformatic analysis involved Phylogenetic tree construction and the sequences of identified phylogenetic neighbors (aligned with the sequences of representative strains using clustal W in built) were applied via Molecular Evolutionary Genetic Analysis Software (MEGA version X). After that, the 16S rRNA gene sequence of the selected *Streptomyces* isolate used in this study has been recorded and deposited in the GeneBank nucleotide sequence database under the accession number PQ416578, and the isolate was highly relatedness to *Streptomyces griseobrunneus.*

#### Optimization process and statistical analysis

Investigating the different process parameters to improved LLO production by *S. griseobrunneus* was considered the main target of our study. Therefore, the impactful nutritional and environmental conditions that determined via Factor-At-A-Time were selected for further statistical optimization using advanced program, Response Surface Methodology (RSM). Since, based on single-factor preliminary experiments, L-lysine (1–6 g/L), MnCl₂ (1–8 g/L) and Ammonium nitrate (4–9 g/L) concentrations significantly affected enzyme activity within, and, so 1–5 g/L, 2–7 g/L, and 5–8 g/L, respectively was selected for subsequent RSM optimization. In this regard, design of Box-Behnken experiment was conducted to obtain a quadratic model based on four independent variables involving, Incubation period (*X1*), L-lysine concentration (*X2*), MnCl_2_ concentration (*X3*), and Ammonium nitrate concentration (*X4*). Four-factor response-surface method for LLO was developed with twenty-seven runs (Table 1). The designation of the targeted model was based on maximum and minimum range for each selected condition and determined the quadratic effects and central points to measure the pure process variability with LLO production as the responses (Y). Generation of the new Minitab 17-software (version 17.0.0) program has been taking place; the potential of this technique was confirmed by the predicted responses resulting from RSM, which were compared with the actual responses. An analysis of variance (ANOVA) using the F-test (a value of *p* < *0.05* was considered significant), defined as the ratio between the pure error square and the lack of fit square was used to evaluate the significance level of the lack of-fit and evaluate the accuracy of this model. Additionally, the Coefficient of determination R^2^ was evaluated which must be close to 1 to determine the significant differences between variables and investigate the efficacy of the designed model.

**Table 1:** Range and levels of experimental variables

Factor	Name	Minimum	Maximum
*X1*	Incubation period (day)	1	5
*X1*	L-lysine concentration(g/L)	1	5
*X3*	MnCl_2_ concentration(g/L)	2	7
*X4*	Ammonium nitrate concentration (g/L)	5	8

### Validation of the results

Subsequently, to validate the obtained results, the response optimizer’s optimum parameters were experimentally conducted and compared to the theoretically expected outputs. The optimized parameters were performed in three replicates, and the results were expressed as mean ± standard deviation.

#### Purification of LLO

##### Chromatography on Sephacryl S-300 column

The initial crude extract was subjected to fractionation via size-exclusion chromatography. This was carried out by a Sephacryl S-300 column (1.75 × 142 cm), which was first equilibrated with 20 mM sodium phosphate buffer, pH 7.0. The separation process used a constant flow rate of 30 mL per hour, and eluted fractions of 2 mL volume were gathered for subsequent analytical steps.

##### Electrophoretic analysis

The purified LLO was analyzed by native PAGE (7% gel) using Smith (1969) method [29], and by denaturing SDS-PAGE (12% gel) according to Laemmli, (1970) [30]. A standard protein ladder was included to estimate molecular mass based on the principles established by Weber and Osborn, (1969) [31]. Proteins were revealed by staining with 0.25% Coomassie Brilliant Blue R-250, after which the gels were destained to remove background dye and enhance band resolution for accurate analysis.

##### Protein quantification

Protein content was measured with the Bradford assay (Bradford, 1976). Following a 10 min. incubation of samples with the reagent at room temperature, absorbance was recorded at 595 nm. Sample concentrations were derived from a standard curve prepared with bovine serum albumin (BSA).

#### Characterization of the purified enzyme

##### Effect of pH on LLO activity and stability

The purified enzyme’s pH optimum and stability profile were characterized across a broad pH spectrum (3.6–9.2) using 20 mM buffers: sodium acetate, sodium phosphate and Tris–HCl pH (3.6–5.6), (5.7–7.0), and (7.2–9.2) respectively. For the stability assay, LLO was pre-incubated in each buffer for one hour at room temperature prior to residual activity measurement. All assays were performed in triplicate under standard conditions.

##### Effect of temperature on LLO activity and stability

The optimum temperature of LLO was determined by measuring catalytic activity over a broad range (20 to 80 °C). A separate thermal stability study was performed by pre-incubating the enzyme solution at defined temperatures (20, 30, 40, 50, 60, 70, and 80 °C) for varying time intervals from 15 to 60 min. After each interval, samples were retrieved, immediately cooled to stabilize the enzyme, and subsequently analyzed for persistent activity using the established assay protocol. This experimental design ensured all determinations were based on triplicate readings.

##### Substrate specificity assay

The specificity of substrate for purified LLO was evaluated against 19 different L-amino acids. The standard assay conditions were modified by replacing the typical substrate, L-lysine, with 5 mM of one of the following test substrates: L-glycine, L-histidine, L-alanine, L-valine, L-tyrosine, L-serine, L-proline, L-cysteine, L-threonine, L-aspartic acid, L-leucine, L-asparagine, L-glutamine, L-tryptophan, L-arginine, L-isoleucine, L-phenylalanine, L-glutamic acid, or L-methionine. Each 100 µL reaction, conducted in 0.05 M Tris–HCl buffer pH 8.0 with 2 mM OPD and 0.81 U/mL HRP, was initiated by adding 20 µL of enzyme. After a 1-h incubation at 37 °C, reaction was stopped by using 2 M H₂SO₄. The activity observed with L-lysine was defined as 100%, and all other activities were expressed as a percentage of this value. All assays, including negative controls lacking either enzyme or substrate, were performed in triplicate.

##### Effect of cations and inhibitors on LLO activity

The impact of cations and inhibitors on activity of LLO was assessed. The enzyme was exposed to each compound (2–5 mM) for 15 min at 25 °C before initiating the standard assay. Tested cations included CaCl₂, CoCl₂, CuCl₂, FeCl₂, MgCl₂, MnCl₂, NiCl₂, and ZnCl₂. Inhibitors from several classes were evaluated: metal chelators (EDTA, 1,10-phenanthroline), reductants and alkylating agents (DTT, β-mercaptoethanol, iodoacetamide), PMSF, KCN, NaN₃, SDS, and K₂Cr₂O₇. Residual activity, determined spectrophotometrically, is reported as mean percentage ± SD relative to untreated control, with all experiments performed in triplicate.

##### Kinetic characterization of LLO

The apparent kinetic parameters for LLO, the maximal velocity (*Vmax*) and the Michaelis constant (*K*_*m*_), were determined by assaying initial velocities at various concentrations of its substrate, L-lysine, under standard conditions. The resulting reaction rates were graphed as a function of substrate concentration to produce Michaelis–Menten plots. These curves were then transformed into linear double-reciprocal plots according to the Lineweaver–Burk method to calculate the kinetic values. Each point is mean of three measurements.

#### Antibacterial activity of the prified LLO

The purified LLO was subsequently subjected to the antibacterial susceptibility in comparison to the crude enzyme and standard antibiotic drugs. Clinically important bacterial pathogens that were used throughout this study were kindly donated from Microbiology and Immunology Dep., Faculty of medicine (Boys), Al-Azhar University. Different bacterial strains such as *Staphylococcus aureus* MRSA (SAM556), *Enterococcus faecalis* (EF733) (Gram-positive), *Escherichia coli* (ECO809) and *Salmonella typhimurium* (ST194) (Gram-negative) were isolated from immunocompromised patients and characterized based on CLSI protocol precautions [32]. A notable virulence of some tested pathogens was sharply indicated by the antibiotic sensitivity profile (Table 2), which reached more than 50% of the resistance ratio for all tested antibiotic agents.

**Table 2:** Antibiotic profile of the bacterial pathogens

Bacterial Pathogen	Antibiotic reference (R/S)										No. of Resistance	% Of Resistance
	E	K	VA	A	CL	CI	PB	G	DA	RF		
*Enterococcus faecalis*	R	R	R	R	R	S	S	S	R	R	7	70
*Staphylococcus aureus MRSA*	R	R	S	R	R	S	S	S	R	S	5	50
*Escherichia coli*	R	R	S	R	R	S	S	S	S	R	5	50
*Salmonella typhimurium*	S	R	S	R	R	S	S	S	S	R	4	40

**E**– Erythromycin (macrolide), **K**– Kanamycin (aminoglycoside), **VA**– Vancomycin (glycopeptide), **A** Ampicillin (penicillin beta-lactam), **CL**- Cephalexin (cephalosporin), **CI-** Ciprofloxacin (fluoroquinolone), **PB**- Polymyxin B (polypeptide), **G**– Gentamicin (aminoglycoside), **DA**- Clindamycin (lincosamide), **RF**- Rifamycin (macrolactams)

Proliferation of each bacterial pathogen could be performed using Mueller Hinton broth medium at 37 °C for 24 h. under shaking conditions. Justification of the inoculum density was approximately performed at 10^6^/mL to keep the inoculum size constant. Subsequently, screening of the purified and crude LLO was implemented at a fixed concentration of LLO (5 U/mL) which was selected based on the pre-screening against the tested pathogens using the turbidometric procedure. The agar well diffusion method was prepared using a Mueller Hinton Agar plate, and each bacterial pathogen was swapped over the plate surface. Adding LLO into the previously prepared well was implemented and subjected to the incubation period individually. Moreover, standard antibiotic agents against microbial pathogens were also applied as a positive control (20 µg/mL) according to the CLSI protocol. Incubation of each tested pathogen was carried out, and the observed inhibition zone diameters (mm) were measured in three replicates and compared to the standard antibacterial agents [33].

##### Determination of MIC values

Accordingly, the Minimum Inhibition Concentration (MIC) test for the purified LLO was prepared against each bacterial pathogen. In this regard, the broth micro dilution method was used based on CLSI [34], since a known weight of LLO was dissolved in sterilized bi-distilled water to prepare a stock solution. Briefly, bacterial strains were grown overnight on Mueller–Hinton agar (MHA), and colonies were suspended in sterile saline to match a 0.5 McFarland standard (≈1 × 10⁸ CFU/mL). The suspension was then diluted in Mueller–Hinton broth (MHB) to obtain a final inoculum concentration of approximately 5 × 10^5^ CFU/mL.

Dilution of LLO was carried out using the broth medium (i.e. Mueller Hinton Broth) to obtain the desired concentration (0.5–20 U/ml). After the incubation period, measurement of the bacterial turbidity was conducted along with taking 10 µL of treated bacterial cell and diluting with sterile distilled water and swapping it onto nutrient agar plates. Enumeration of the Colony Forming Unit (CFU) was performed after 24 h. and MIC was also determined. Determination of MIC value for each targeted compound is known as the lowest concentration of each sample that yields a minimum number of CFU compared to the untreated samples [35].

## Results and discussion

### Screening of the marine *Streptomyces* isolates for LLO production

The ability of the *Streptomyces* isolates to produce LLO in the presence of the inducer agent (L-lysine) was investigated qualitatively and quantitively. In this experiment, twenty-two *Streptomyces* isolates were investigated by cultivating them individually in a medium supplemented with L-lysine for 4 days and the resulting supernatant was used for the detection of the crude enzyme. The efficiency of the tested isolates to secrete LLO was initially evaluated qualitatively, which demonstrated the potentiality of some isolates to produce LLO at varied ratios. A comparative study between the LLO-producing isolates was also determined in the supernatant of the isolates, which deeply showed the most potent producers (Table 3). In addition, the production of LLO was greatly differentiated among the tested *Streptomyces* isolates, with the maximum LLO production being established by isolates S3, S15, and S19. Interestingly, the highest productivity of LLO was obtained by isolate S15 (8 U/mg). From our findings, we can conclude that the marine *Streptomyces* isolate S15 was found to be the most potent producer, which obtained a maximum LLO after 96 h. of incubation. Thus, further studies were performed to optimize the LLO productivity.

**Table 3:** Screening of the marine *Streptomyces* isolates for LLO production

Isolates code	LLO Specific activity (U/mg)
1	0.09 ± 0.03
2	0.05 ± 0.01
3	3.9 ± 0.22
4	0
5	0.77 ± 0.01
6	0
7	0
8	0.12 ± 0.05
9	0
10	0
11	0
12	0
13	0
14	0
15	8.02 ± 0.41
16	0
17	0.18 ± 0.12
18	0
19	6.1 ± 0.71
20	0
21	0
22	1.9 ± 0.07

### Identification of marine *Streptomyces* by 16S rRNA gene sequence analysis

**Fig. 1:**
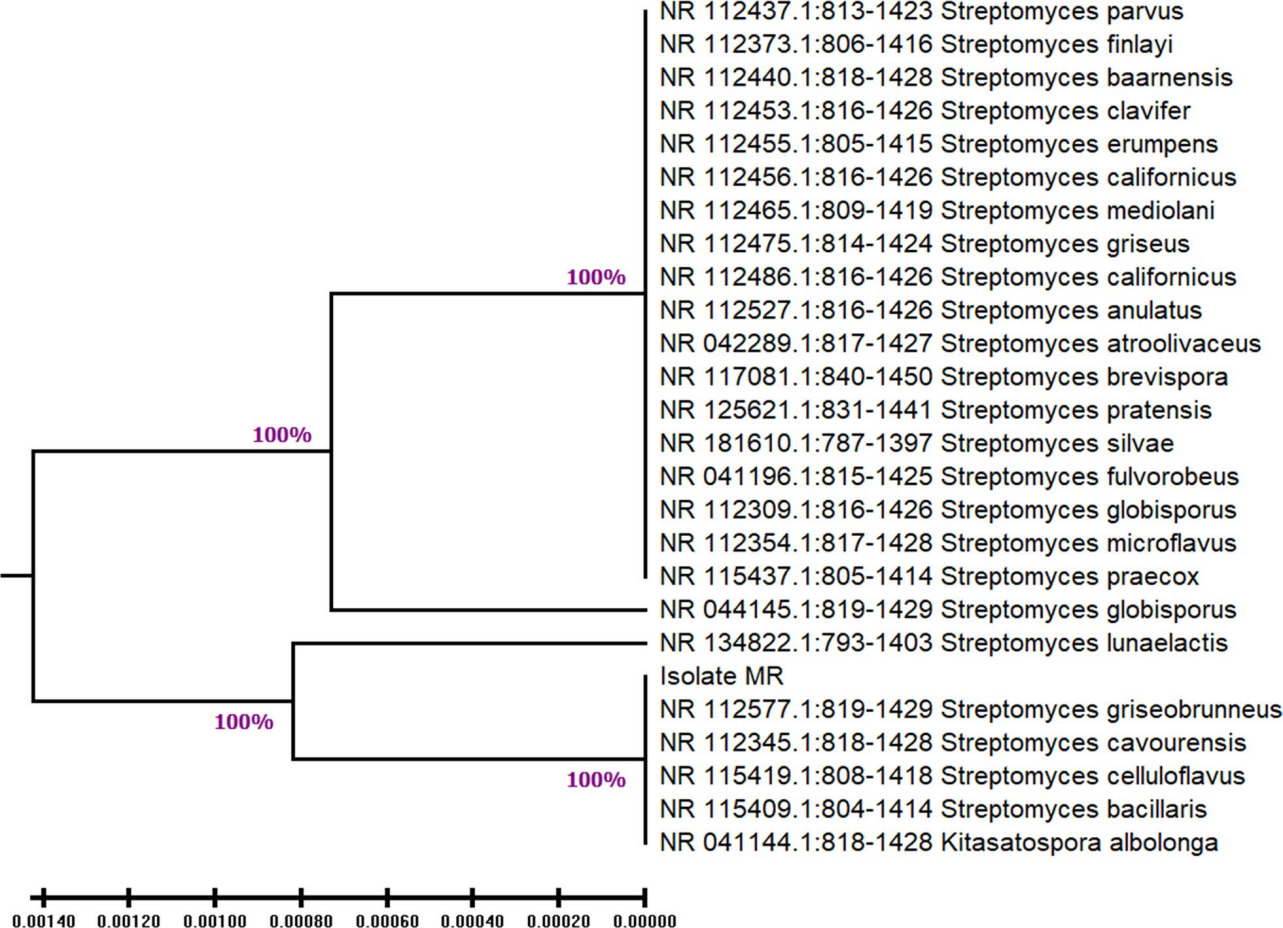
Phylogenetic tree based on partial 16S rDNA sequences, showing the relationship between isolate *Streptomyces griseobrunneus (*PQ416578) strain MR and other species belonging to the genus *Streptomyces*

Molecular identification of the most potent LLO-producing isolate S15 has been applied employing the genetic characterization that is based on 16S rRNA gene analysis; PCR amplification of the 16S rRNA gene was conducted using universal primers. Results showed that the highest similarity of the targeted sequence of 16S rRNA gene with *Streptomyces* strain was up to 99%. In this regard, 16S rRNA sequences from the *Streptomyces* species were obtained from the NCBI (www.ncbi.nlm.nih.gov/) and a phylogenetic tree was constructed by the neighbour-joining method (Fig. 1). The molecular analyses suggested that the isolate S15 was closely related to *Streptomyces griseobrunneus* strain. Therefore, it was identified as *Streptomyces griseobrunneus* S15 and recorded in GenBank under accession number, PQ416578.

### Optimization process and statistical analysis

Cultivation of *Streptomyces griseobrunneus* S15 under different nutritional and environmental conditions was performed to determine the significant ones and then they were optimized by the RSM approach. Optimization of the significant parameters by RSM procedure and verification was carried out by Minitab program. Optimization by Box-Behnken design was applied to gather detailed information about some factors that were found to have a major impact on performance during screening. Finally, the validation of the results could be checked by verification experiments under the predicted optimized conditions.

### Response surface experimental approach using Box-Behnken design

Selection of four independent parameters in the optimal range and generated via Box-Behnken was implemented with 27 run orders for LLO yield at 30 °C under shaking condition. Specific activity of LLO was represented as the response value (Y). As shown in Table 4, greatest differences in productivity were clearly noted between different runs, which reflected the significance of the targeted conditions at different levels. The results indicated a variation in LLO productivity, ranging from 11.7 to 42.9 U/mg, highlighting the distinctive effectiveness of the designated program in achieving maximum production efficiency.

**Table 4:** Optimization of the LLO production by *Streptomyces griseobrunneus* S15 via Response Surface Methodology (RSM), Box-Behnken Design

Run no	*X1:* *Incubation period*	*X2:* *L-lysine*	*X3:* *MnCl*_*2*_	*X4:* *Ammonium nitrate*	*Y*: L-Lysine oxidase Specific activity (U/mg)	
					**Observed**	**Predicted**
1	7	0.2	0.2	0.4	13.9	12.9583
2	7	0.05	0.2	0.4	42.9	40.6917
3	5	0.125	0.1125	0.2	28.4	32.8167
4	7	0.05	0.1125	0.2	17.9	19.4000
5	9	0.2	0.1125	0.4	25.2	21.8083
6	7	0.125	0.025	0.2	40.0	36.7833
7	9	0.125	0.025	0.4	25.0	23.3250
8	7	0.125	0.1125	0.4	29.0	31.2250
9	7	0.2	0.1125	0.6	31.9	31.4750
10	7	0.125	0.1125	0.4	18.6	17.9667
11	7	0.125	0.1125	0.4	11.7	11.8583
12	7	0.2	0.025	0.4	34.7	34.5417
13	7	0.125	0.025	0.6	41.7	40.9000
14	9	0.125	0.1125	0.2	14.2	17.3083
15	5	0.125	0.1125	0.6	40.2	40.9000
16	7	0.125	0.2	0.6	23.8	25.7250
17	9	0.05	0.1125	0.4	30.0	30.6583
18	5	0.125	0.025	0.4	18.7	20.5583
19	7	0.05	0.025	0.4	33.3	35.3833
20	7	0.2	0.1125	0.2	20.8	24.8833
21	5	0.2	0.1125	0.4	40.8	40.9000
22	7	0.125	0.2	0.2	38.5	39.5917
23	9	0.125	0.2	0.4	29.0	28.2583
24	5	0.05	0.1125	0.4	38.1	35.5917
25	7	0.05	0.1125	0.6	37.9	36.6833
26	9	0.125	0.1125	0.6	15.6	12.0500
27	5	0.125	0.2	0.4	28.8	26.3583

As can be seen in Table 5, analysis of variance (ANOVA) referred to the significant parameters in our data. The ANOVA of LLO production showed the significance of the model, with the model F-value of 2.94, i.e., (Prob > F) which was less than 0.05. The values of “Prob > F of less than 0.05 indicate that the model terms are significant. Furthermore, the ANOVA of the model for LLO reflected that it fits well because the “Lack of Fit F-value” was 1.35, indicating that the Lack of Fit is not significant in relation to the pure error. Besides, the regression coefficient (R^2^) was utilized to obtain the fit of the model. It was shown that the model regression coefficient (R^2^) was found to be near 0.84 for LLO demonstrating that all the points of the model can be predicted. The predicted model R^2^ was 0.84, which is considerably close to the adjusted R2 of 0.68. Indeed, the convergence between the predicted and adjusted R2 values could refer to the model fitness, which is in a reasonable agreement with the model adequate precision.

**Table 5:** Analysis of variance (ANOVA)

Source	DF	Adj SS	Adj MS	F-Value	P-Value (Prob > F)
Model	14	8890.8	635.06	2.49	0.024
Linear	4	2849.3	712.33	2.79	0.055
Incubation period	1	175.8	175.8	0.69	0.423
L-lysine	1	153.4	153.44	0.6	0.403
MnCl_2_	1	289.7	289.69	1.13	0.193
Ammonium nitrate	1	2230.4	2230.41	8.74	0.012
Square	4	4868.6	1217.16	4.77	0.016
Incubation period*Incubation period	1	3019.6	3019.64	11.83	0.005
L-lysine*L-lysine	1	78.7	78.66	0.31	0.589
MnCl2*MnCl_2_	1	54.3	54.33	0.21	0.653
Ammonium nitrate*Ammonium nitrate	1	195.2	195.16	0.76	0.099
2-Way Interaction	6	1172.8	195.47	0.77	0.611
Incubation period*L-lysine	1	114.4	114.38	0.45	0.516
Ammonium*MnCl_2_	1	181.6	181.58	0.71	0.415
Incubation period*Ammonium nitrate	1	739.6	739.57	2.9	0.054
L-lysine*MnCl_2_	1	31.9	31.92	0.13	0.73
L-lysine*Ammonium nitrate	1	22.5	22.47	0.09	0.772
MnCl_2_*Ammonium nitrate	1	181.6	181.58	0.71	0.415
Error	12	3062.8	255.23		
Lack-of-Fit	10	2668	266.8	1.35	0.498
Pure Error	2	394.8	197.42		
Total	26	11953.6			

R^2^ = 0.843; Adj R^2^ = 0.688. df degrees of freedom

The regression equations of LLO as follows:

#### Regression equation in terms of actual factors

$$ \begin{gathered} L - ly\sin e \, oxidase \, \left( {U/mg} \right) = - 162.2 + 5.74 X1 \hfill \\ + 0.32 X2 + 53.83 X3 \hfill \\ + 1.238 X4 - 0.813 X1*X1 \hfill \\ - 0.656 X2*X2 - 4.656 X3*X3 \hfill \\ - \, 0.01270 X4*X4 \hfill \\ + 0.063 X1*X2 + 0.250 X1*X3 \hfill \\ - 0.0469 X1*X4 + 0.688 X2*X3 \hfill \\ - 0.0156 X2*X4 - 0.0078 X3*X4. \hfill \\ \end{gathered} $$

According to the ANOVA results (Table 4), the P value of the regression model is < 0.005. This indicates the regression equation was used to express the relationship between each variable and response value demonstrating a significant linear relationship between the dependent variable and each independent variable.

**Fig. 2:**
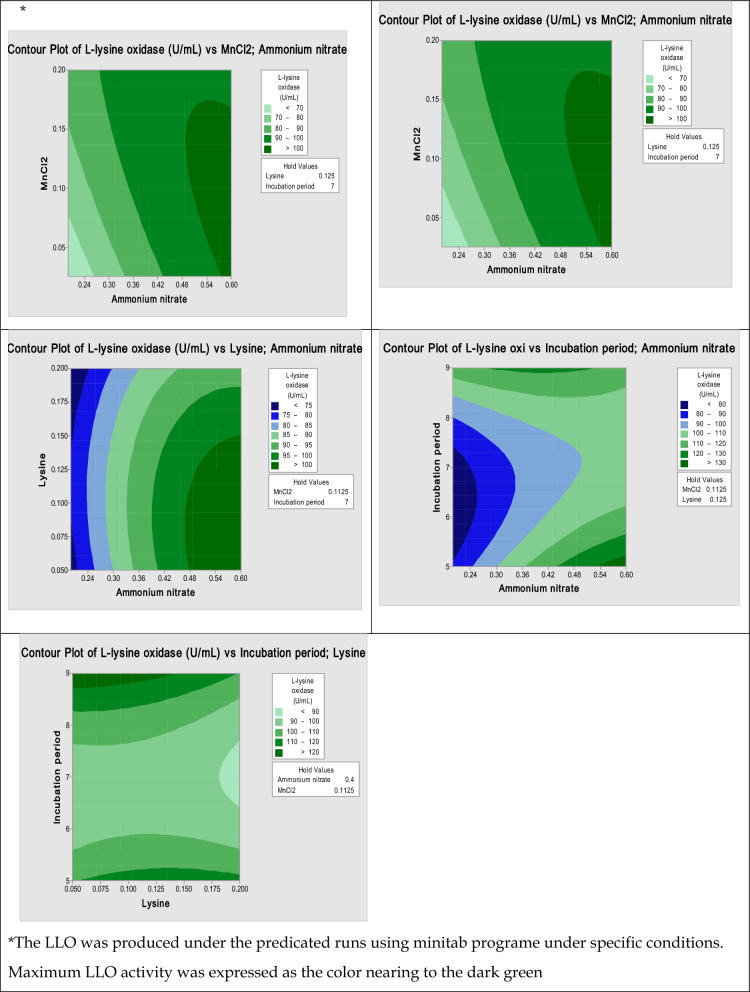
Contour line plot of the effect of cross-interaction among different variables on LLO by *Streptomyces griseobrunneus* S15

To investigate the response surface shape, the analysis chart was generated based on the regression equation. The response surfaces profiles between each parameter (for each model) are all open downward along with a convex direction, indicating high LLO production (Fig. 2). Additionally, the contour centers of the three response surfaces fall within the set range, referring to optimal conditions of the model occurring on the level of designed factors. The response surface is steep, reflecting the significant effect of Ammonium nitrate and L-lysine on enzyme production (Fig. 2).

### Validation of the results

**Fig. 3:**
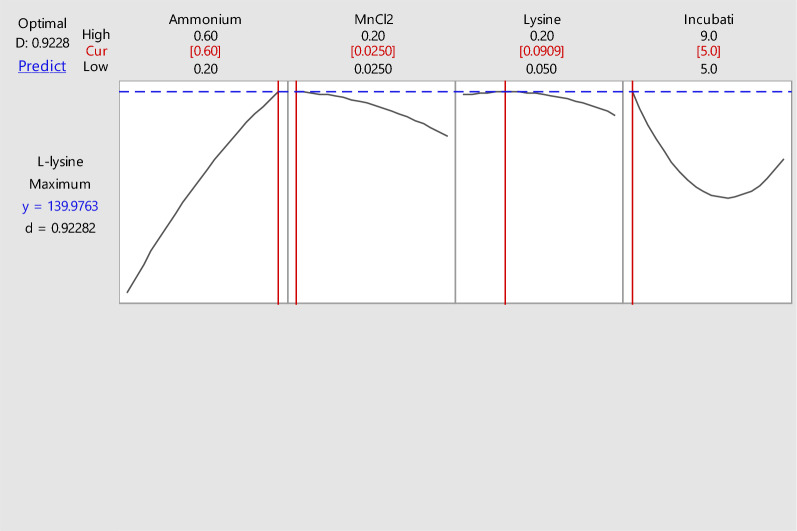
Response optimizer for LLO (U/ mL) production by *S. griseobrunneus* S15

To confirm the validity of the targeted program, a predicated condition was generated and investigated after analysis of the obtained results. Accordingly, three different runs included the predicated conditions with a desirability of 1.0 and maximum LLO productivity were determined (Fig. 3). In comparison, the theoretical values were found to be in consistence with the practical values for which the enzymatic activity was 133 U/mL. Thus, the compatibility of theoretical and practical results reflected the validity of the designated model. Accordingly, the optimized parameters that could induce a significant increase in enzymatic activity (133 U/mL) were as follows: 0.6% ammonium nitrate, 0.2% MnCl_2_, 0.2% L-lysine concentrations and 9 days of the cultivation period, which was nearing the predicted value (139.9 U/mL).

### Purification of LLO from *Streptomyces griseobrunneus* S15

**Fig. 4:**
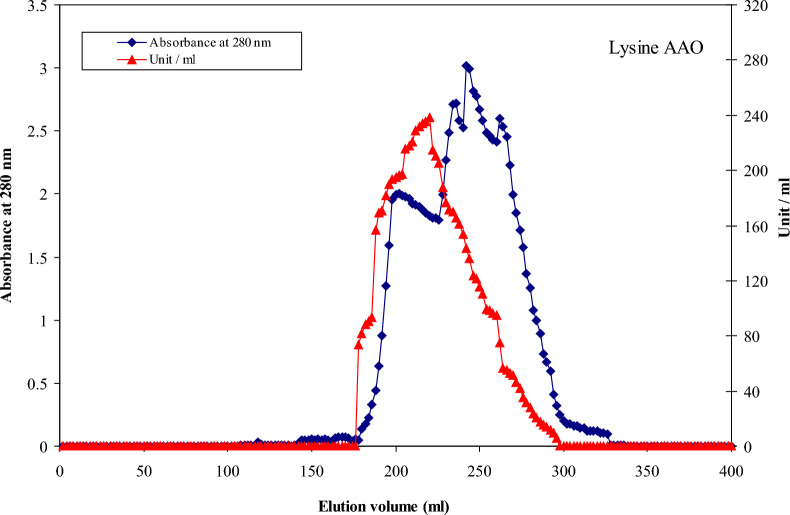
A chromatographic typical elution profile for LLO crude extract on Sephacryl S-300 column

The purification of LLO from *Streptomyces griseobrunneus* S15 was accomplished efficiently in a single gel filtration step, which resolved a distinct active peak (Fig. 4) and enhanced the specific activity from an initial 668.67 U/mg to a final 1575.9 U/mg. This represents a 2.356-fold purification with a yield of 49.99% (Table 6). The eluted LLO showed a single, symmetrical well-resolved peak, indicating effective resolution. Enzymatic activity was detected within a retention volume range of 178–262 mL, with maximal activity centered at approximately 220 mL. The corresponding elution time extended from 5 h 55 min to 8 h 45 min, reaching a maximum at 7 h 20 min. Fractions collected across this peak region (generally fractions 89–131), particularly around fraction 110, demonstrated the highest specific activity. A comparative analysis with existing literature reveals high specific activity of our preparation. Whereas Kusakabe et al. (1980) [15] reported a specific activity of 66 U/mg for *T. viride* LLO, and Smirnova et al. (2025) [12] achieved 292 U/mg for the *T. harzianum* enzyme, our value of 1575.9 U/mg is significantly greater. Although the yield reported by Smirnova et al. [12] (78%) was higher, the paramount catalytic efficiency achieved by our simplified protocol highlights its effectiveness for producing highly active LLO.

**Table 6:** A typical purification scheme of LLO from *Streptomyces griseobrunneus* S15

Purification steps	Total protein (mg)	Total Activity (unit)	Specific Activity (units/mg)	Yield (%)	Purification (fold)
Crude extract	14.75	9863	668.67	100.0	1.000
Sephacryl S-300 L-lysine α-oxidase (LLO)					
	3.129	4931	1575.9	49.99	2.356

One unit of LLO activity was defined as the amount of enzyme required to produce 1 µmol of H_2_O_2_ per minute under the conditions described.

**Fig. 5:**
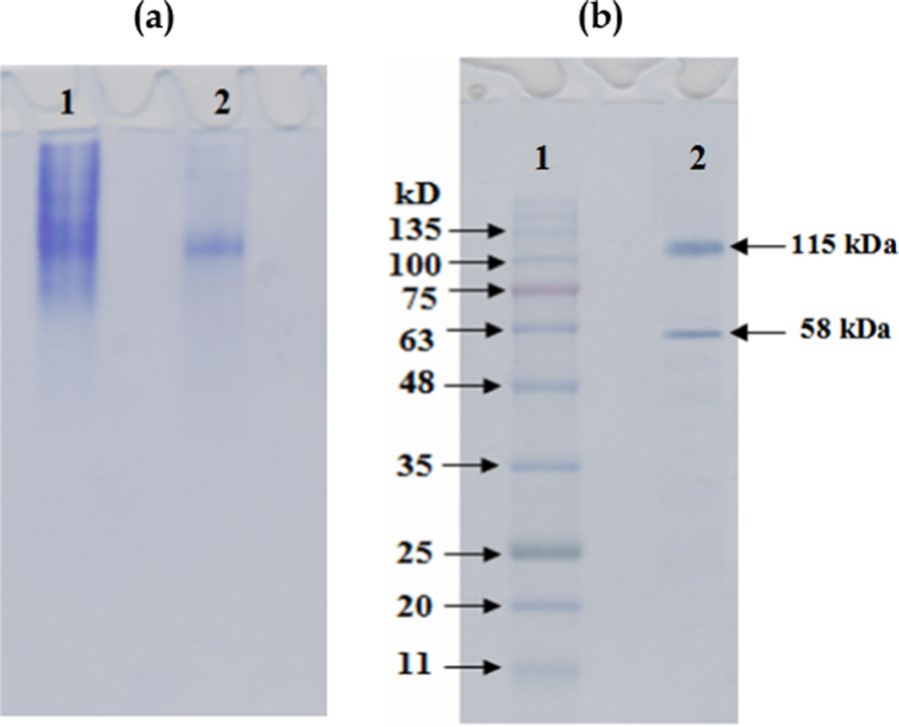
**a** Electrophoretic analysis of LLO pattern of the different purification steps on 7% native polyacrylamide gel: (1) crude enzyme (2) Sephacryl S-300 fraction **b**: Subunit molecular weight determination by electrophoretic analysis of purified LLO on 12% SDS–polyacrylamide gel: (1) Marker proteins and (2) Pure LLO; native molecular weight: 115 kDa; subunit molecular weight: 58 kDa

Electrophoretic techniques were employed to determine the molecular weight and purity of LLO. The final preparation showed a single band on native (7% gel) (Fig. 5a, b), confirming its homogeneity. The native molecular weight was estimated at approximately ~ 115 kDa by Sephacryl S-300 column gel filtration, while SDS-PAGE revealed a subunit size of approximately ~ 58 kDa, suggesting a homodimeric structure. This result is consistent with previous reports on microbial LLOs, which are typically ~ 115 kDa homodimers composed of ~ 58 kDa subunits (e.g., Pokrovsky et al., 2013 [24]; Weber et al., 1994 [13]; Isobe et al., 2007 [16]). The congruence of our structural data with the literature underscores the reliability of our purification method. The rationale for employing a single chromatographic step was based on the high selectivity of gel filtration for the target protein’s molecular size (~ 115 kDa homodimer) and the observation of a distinct, symmetrical activity peak corresponding to the target enzyme (Fig. 4). This strategy prioritized maximizing yield and catalytic activity recovery for subsequent characterization and antibacterial assays. While additional purification steps could potentially enhance homogeneity, the single-step process yielded a preparation of sufficient purity for reliable kinetic and functional analysis, as evidenced by a single band on native PAGE (Fig. 5a) and a clear, single subunit band on SDS-PAGE (Fig. 5b). The Coomassie-stained LLO protein band confirmed that the observed protein was free of other contaminating proteins.

### Characterization of LLO from *Streptomyces griseobrunneus*

#### Optimum pH and pH stability of LLO

**Fig. 6:**
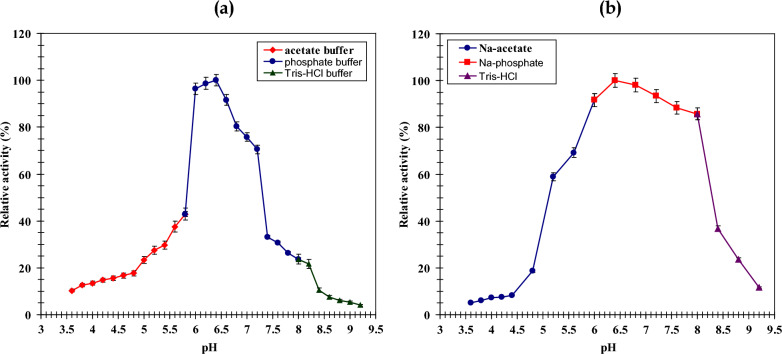
**a** The optimum pH for activity of purified LLO using 20 mmol L-1 (Na-acetate, Na-phosphate and Tris–HCl bufferpH) (3.6 to 5.6), (5.7 to 7.0), (7.2 to 9.2) respectively. **b** pH stability for LLO activity using buffers of different pH

The catalytic efficiency of the enzyme was strongly influenced by pH, with considerable variation observed across the tested buffer systems—acetate, phosphate, and Tris–HCl. Peak activity, designated as 100%, occurred specifically in phosphate buffer at pH 6.4, establishing this as the optimum for LLO function (Fig. 6a). A pronounced reduction in activity was evident in both acetate and Tris–HCl buffers, implying that the ionic composition of phosphate buffer is uniquely conducive to the reaction. A sharp decline in performance was noted at acidic and alkaline extremes, where activity fell below 10% at pH values under 5.0 or over 9.0. The identified pH optimum corresponds with earlier characterizations of LLO from *Trichoderma* spp., which also display peak function at neutral to mildly alkaline pH values [36]. Comparable behavior was documented for a *Pseudomonas* sp. AIU 813 LLO, exhibiting maximal activity at pH 7.0 alongside a broader functional range [16]. Conversely, LLO isoforms from *Trichoderma cf. aureoviride* and *T. harzianum* demonstrate distinct profiles, with activity optima at pH 8.0 [14, 24] and sustained high activity within the pH 8.0–9.0 range [37], respectively. These organism-specific profiles underscore how microbial source dictates enzymatic characteristics, likely a result of evolutionary adaptation to native habitats. Stability assessments revealed the enzyme maintains integrity near physiological pH, preserving over 90% of its activity after incubation in phosphate buffer at pH 7.5 (Fig. 6b). This pronounced stability under neutral conditions indicates a potential suitability for applications within physiological systems such as human blood. In contrast, exposure to acidic or alkaline conditions (pH 5.2 and 9.0) severely diminished stability, reducing residual activity to less than 25%. The correlation between its pH stability (pH 7.0–7.5) and peak activity (pH 6.4) confirms its neutral preference, a well-established trait common among microbial LLOs [16, 24].

### Optimum temperature and thermostability of LLO

**Fig. 7:**
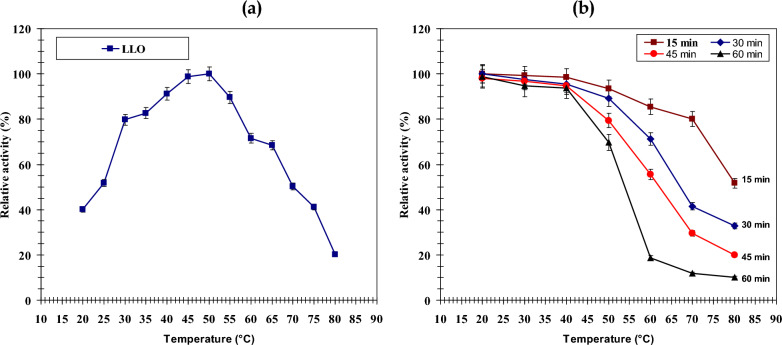
**a** The temperature optimum for purified LLO activity at different temperature (20—80 °C). **b** thermal stability of purified LLO at various temperatures (30°- 40°- 50°- 60°- 70°- 80 °C) for a range of periods (15, 30, 45 and 60 min)

A characterization of the temperature optimum for the *Streptomyces griseobrunneus* LLO revealed a marked influence of heat on its function. Catalytic activity intensified progressively from 20 °C until reaching a maximum at 50 °C, which was assigned a value of 100% (Fig. 7a). Beyond this peak, a severe reduction occurred, yielding only 20.3% relative activity at 80 °C. This predictable parabolic response is typical of enzymes susceptible to heat, illustrating the equilibrium between enhanced reaction kinetics and the onset of protein denaturation. The determined optimum of 50 °C corresponds well with values published for LLOs derived from other microorganisms. Isoforms from *Trichoderma harzianum* and certain *Streptomyces* species display analogous peaks within the 40–50 °C interval [37]. Certain fungi, however, produce more heat-resistant variants; for example, the LLO from *Penicillium steckii* AIU 027 is most active at 55 °C [16]. This diversity in thermal profiles among origins points to adaptive evolution within distinct habitats. The recurring tendency for optima to fall within a 40–55 °C window implies an inherent structural limitation for this enzyme family, whereas outliers like the *P. steckii* LLO highlight possibilities for enhancing heat resistance through protein engineering. Investigations into the enzyme’s stability at various temperatures showed it maintains more than 90% of its initial activity following a one-hour incubation at 40 °C or below (Fig. 7b). Its stability diminished at warmer conditions, preserving 69.7% activity after one hour at 50 °C and falling below 20% at 60 °C. This swift deactivation at extreme heat implies a loss of native structure, a recognized trait of enzymes from mesophilic organisms. Importantly, the thermal endurance of the *S. griseobrunneus* LLO proved superior to that of enzymes isolated from *Trichoderma cf. aureoviride* Rifai VKMF4268D and *Trichoderma harzianum* [14, 37], suggesting a more resilient conformation.

### Substrate specificity of purified LLO

The substrate range of the L-lysine oxidase (LLO) was evaluated using a series of L-amino acids. The enzyme demonstrated pronounced selectivity, displaying its highest catalytic efficiency with its native substrate, L-lysine, which was set at 100% relative activity (Table 7). Significant activity was also recorded for L-arginine (91.9 ± 2.24%), L-leucine (83.74 ± 2.10%), L-glycine (82.69 ± 1.75%), and L-phenylalanine (81.15 ± 1.88%), indicating a capacity to accommodate residues with basic, aliphatic, and aromatic side chains. Conversely, oxidation rates fell below 2% for L-histidine and were undetectable for L-valine and L-proline, implying stringent steric or electrostatic limitations within the catalytic site. Moderate oxidation levels, ranging from 61.98% to 77.13%, were measured for polar (L-serine, L-threonine) and aromatic (L-tyrosine, L-tryptophan) amino acids. Acidic residues, L-aspartate and L-glutamate, proved to be poorer substrates (< 50% activity), potentially due to charge repulsion with the active site environment. The notably low activity toward L-cysteine (9.66 ± 0.45%) could result from inhibitory effects of the thiol group or suboptimal binding. This substrate profile is partially consistent with documented microbial LLO behavior, which commonly favors basic amino acids like L-lysine and L-arginine [16]. However, the pronounced activity against neutral aliphatic amino acids (e.g., L-leucine) represents a distinctive feature, suggesting an unusually flexible active site architecture in this enzyme. This broad specificity could be advantageous for anticancer applications, because it enables the depletion of multiple amino acids essential for tumor growth [12]. This stands in contrast to the narrow substrate specificity reported for LLOs from various *Trichoderma* species, which are predominantly active only on L-lysine [13, 15, 37]. For instance, the LLO from *Trichoderma cf. aureoviride* was found to be exclusive for lysine, showing minimal activity on other substrates like arginine [14]. The expanded amino acid specificity of the *Streptomyces griseobrunneus* LLO may therefore hold significant relevance for its development in biomedical contexts.

**Table 7:** Substrate specificity of *Streptomyces griseobrunneus* LLO

Substrate (5 mM)	Relative activity (%) ± S.D
L-Lys	100.0
L-Gly	82.69 ± 1.75
L-His	1.76 ± 0.08
L-Ala	88.81 ± 2.43
L-Val	0.00
L-Tyr	77.13 ± 1.96
L-Ser	68.15 ± 2.32
L-Pro	0.00
L-Cys	9.66 ± 0.45
L-Thr	65.95 ± 2.14
L-Asp	48.98 ± 1.63
L-Leu	83.74 ± 2.10
L-Asn	11.37 ± 0.52
L-Gln	14.29 ± 0.66
L-Trp	61.98 ± 1.95
L-Arg	91.90 ± 2.24
L-Ile	26.58 ± 1.02
L-Phe	81.15 ± 1.88
L-Glu	45.26 ± 1.47
L- Met	79.06 ± 1.90

### Effects of cations and inhibitors on LLO activity

Divalent metal cations function as vital cofactors for numerous enzymes, contributing to structural integrity, catalytic function, and overall stability. To assess these effects on *Streptomyces griseobrunneus* LLO, its activity was measured following exposure to various cations and inhibitors. The enzyme's response to metal ions was highly variable (Table 8). A substantial activating influence was observed with Mg^2^⁺, which elevated activity by 33.6% and 59.4% at 2 mM and 5 mM, respectively, potentially indicating a stabilizing interaction with the enzyme's three-dimensional form. Mn^2^⁺ also served as a strong activator (11.5% and 45.2% increase), while Ca^2^⁺ induced a slight stimulatory effect (4.8–9.7% increase). Conversely, ions such as Cu^2^⁺ and Fe^2^⁺ acted as powerful inhibitors, suppressing activity by 69.8–80.8% and 76.6–87.2%, likely through oxidative mechanisms or direct blockage of the active site. Moderate suppression was caused by Co^2^⁺ (10.8–23.6% reduction), Ni^2^⁺ (36.3–58.3% reduction), and Zn^2^⁺ (18.6–31.9% reduction), possibly due to competition with native cofactors or structural ions. These findings imply that whereas ions like Mg^2^⁺ and Mn^2^⁺ may act as beneficial cofactors, others—especially Cu^2^⁺ and Fe^2^⁺—markedly disrupt enzymatic function, an important insight for applications involving this oxidase. This cation-inhibition profile is consistent with earlier reports; for example, CuSO₄ and FeSO₄ strongly suppressed LLO from *Aspergillus terreus* [38]. Similarly, Hg^2^⁺ and Zn^2^⁺ inhibited LLO from *Trichoderma harzianum*, while Mn^2^⁺ and Co^2^⁺ enhanced its activity [39]. The inhibitor susceptibility profile further clarified the enzyme’s mechanistic traits (Table 9). Reducing agents DTT and β-mercaptoethanol caused strong inhibition (51–88%), underscoring the importance of disulfide bonds in maintaining functional structure. Moderate inhibition by PMSF and iodoacetamide (30–68%) points to the involvement of serine and cysteine residues in the catalytic mechanism. While EDTA had negligible effect (< 4% inhibition), partial inhibition by 1,10-phenanthroline (20–50%) may indicate limited metal interaction. Notably, the enzyme was largely resistant to SDS (< 8% inhibition) and only weakly sensitive to KCN and NaN₃ (14–30% inhibition), setting it apart from many metallo- or flavoenzymes. This inhibition pattern suggests a catalysis mechanism reliant on redox-active thiols rather than metal ions, with structural stability governed by disulfide bridges. These observations align with previous work; reducing agents are known to disrupt disulfide bonds and inhibit activity, whereas cyanide has been reported to have little effect on certain LLOs, implying no direct interaction with the enzyme’s redox center [39]. The distinct sensitivity pattern may reflect specific evolutionary adaptations in this bacterial oxidase compared to fungal forms.

**Table 8:** Effect of divalent cations on purified LLO

Reagent	Final Concentration (mM)	Residual activity (%) ± S.D
Control	–	100.0
CaCl_2_	2.0	104.8 ± 3.1
	5.0	109.7 ± 2.8
CoCl_2_	2.0	89.21 ± 2.5
	5.0	76.43 ± 2.1
CuCl_2_	2.0	30.18 ± 1.4
	5.0	19.23 ± 0.9
FeCl_2_	2.0	23.45 ± 1.1
	5.0	12.83 ± 0.6
MgCl_2_	2.0	133.6 ± 3.9
	5.0	159.4 ± 4.7
MnCl_2_	2.0	111.5 ± 2.7
	5.0	145.2 ± 3.8
NiCl_2_	2.0	63.75 ± 1.9
	5.0	41.69 ± 1.3
ZnCl_2_	2.0	81.36 ± 2.2
	5.0	68.11 ± 1.9

**Table 9:** Effect of various inhibitors on LLO

Reagent	Final Concentration (mM)	Inhibition (%) ± S.D
Control	–	0.0
Ethylenediamine tetra acetic acid (EDTA)	2.0	1.67 ± 0.05
	5.0	3.85 ± 0.12
DL-Dithiothreitol (DTT)	2.0	51.2 ± 1.5
	5.0	79.6 ± 2.4
β-Mercaptoethanol	2.0	42.3 ± 1.3
	5.0	87.6 ± 2.6
1,10 Phenanthroline	2.0	20.5 ± 0.62
	5.0	49.7 ± 1.5
Phenyl methyl sulfonyl fluoride (PMSF)	2.0	30.6 ± 0.92
	5.0	59.8 ± 1.8
Iodoacetamide	2.0	30.5 ± 0.91
	5.0	67.9 ± 2.0
Potassium cyanide (KCN)	2.0	23.4 ± 0.70
	5.0	29.8 ± 0.90
Sodium azide (NaN_3_)	2.0	13.6 ± 0.41
	5.0	17.8 ± 0.53
Sodium dodecyl sulphate (SDS)	2.0	3.50 ± 0.11
	5.0	7.90 ± 0.24
Potassium dichromate (K_2_Cr_2_O_7_)	2.0	15.6 ± 0.47
	5.0	26.8 ± 0.80

### Kinetic parameters

**Fig. 8:**
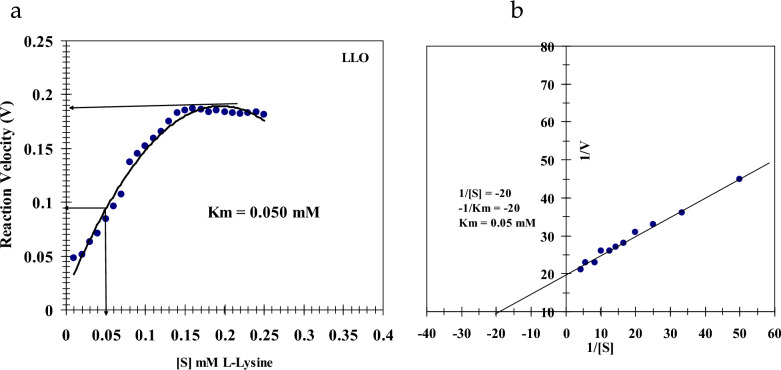
**a** Effect of L-Lysine concentration in mM on purified LLO reaction velocity. **b** Lineweaver–Burk plot relating the reciprocal of purified LLO reaction velocity to L-Lysine concentration in mM

The catalytic kinetics of *Streptomyces griseobrunneus* LLO were characterized using L-lysine as the substrate. The enzyme followed classical Michaelis–Menten kinetics, with initial velocity increasing hyperbolically with rising substrate concentration until reaching a maximum rate, a behavior commonly reported for L-lysine α-oxidases and other L-amino acid oxidases [10, 16, 35]. Analysis of this saturation curve yielded a Michaelis constant (***K***_***m***_) of 0.050 mM and a maximum velocity (*Vmax*) of 6.19 U/mg (Fig. 8a). These kinetic parameters were further validated through linearization of the data using a Lineweaver–Burk double-reciprocal plot, which confirmed the same ***K***_***m***_ value of 0.050 mM and demonstrated strong linear correlation (Fig. 8b). The notably low ***K***_***m***_ value reflects an exceptionally high binding affinity for L-lysine. This strong substrate affinity suggests the enzyme operates efficiently even at minimal substrate concentrations, a particularly advantageous characteristic for therapeutic applications aimed at amino acid depletion. Comparative analysis reveals this affinity is approximately 20% greater than that reported for LLO from *Rhodococcus* sp. AIU Z-35-1, which demonstrated a ***K***_***m***_ of 0.062 mM [10]. The combination of high catalytic efficiency, superior substrate affinity, and previously established stability profiles indicates this bacterial LLO possesses significant potential for development in biomedical applications, particularly in strategies targeting tumor metabolism through selective lysine degradation.

### Antibacterial activity of LLO

**Fig. 9:**
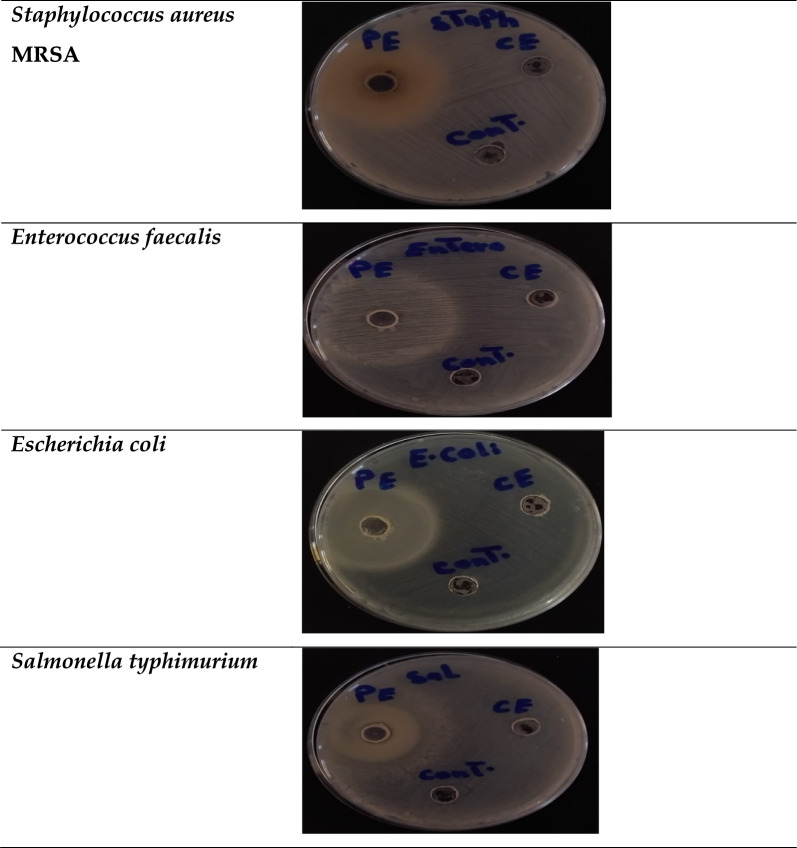
Antibacterial activity of the purified LLO using agar-well diffusion. (PE) purified enzyme, (CE) crude enzyme, (cont.) Phosphate buffer

Drug-resistant bacteria still represented an extreme health problem due to those possessing an adaptive survival mechanism. The rapid distribution of the Multi-Drug Resistance (MDR) bacteria and its ability to avoid the eradicated activity of the antibacterial agents is commonly related to their capability to produce a biofilm matrix. Therefore, discovering new antibacterial agents that contributed to the inhibition process of MDR is considered urgently desirable. In this study, utilization of LLO to treat different MDR bacterial pathogens was initially screened at a fixed concentration of the crude and purified enzyme (Fig. 9). An antibacterial agent (Cephradine), which was resisted by all tested bacterial pathogens was also included in the agar-well diffusion test to demonstrate the ability of those to proliferate even at 20 µg/mL. As shown in Table 10, most of the bacterial pathogens were inhibited in the presence of purified LLO; however, the crude LLA could not observe any activity, the same as Cephradine. The neglected activity of crude enzyme may be attributed to the presence of other proteins that contributed to the decrease of LLO activity. In this respect, the strong inhibition activity of purified LLO was clearly obtained against *E. faecalis* (11 ± 0.27) followed by *E. coli* (9 ± 0.27). In addition, considerable activity of purified LLO was also observed toward *S. aureus* (7 ± 0.05) and *S. typhimurium* (8 ± 0.02). Overall, the targeted LLO demonstrated significant efficacy against all studied bacterial infections, outperforming traditional antibiotic standards. Indeed, L-amino acid oxidase enzymes were extensively reported as bactericidal agents due to the oxidative deamination reaction that mainly triggers hydrogen peroxide [40]. Hydrogen peroxide, an end product of oxidative deamination reaction, is considered a potent antibacterial agent that induces DNA damage and causes cell death through generation of reactive oxygen species (ROS). Accordingly, LLO directly contributes to enhancing the potential eradication strategy toward the bacterial pathogen [6]. LLO from *Trichoderma harzianum* was found to be effective against the highly contagious bacterium *Erwinia amylovora,* with the greatest inhibitory effect obtained at 0.54 U/ml [41]. Furthermore, the ability of *Trichoderma harzianum* LLO to inhibit the phytopathogenic fungi and different bacterial pathogens was also reported [2], the cell lysis was increased with an increasing enzyme dose.

**Table 10:** Antibacterial activity of LLO using agar-well diffusion

L-lysine Oxidase (5 U/mL)	Inhibition Zone (mm)			
	***Staphylococcus aureus*** **MRSA**	***Enterococcus faecalis***	***Escherichia coli***	***Salmonella typhimurium***
Crude enzyme	ND^b^	ND	ND	ND
Purified enzyme	7 ± 0.05	11 ± 0.27	9 ± 0.27	8 ± 0.02
Ciprofloxacin^a^	4 ± 0.27	3 ± 0.27	4 ± 0.06	4 ± 0.21
Cephradine	ND	ND	ND	ND
Erythromycin	ND	2 ± 0.05	ND	ND
Polymyxin B	2 ± 0.27	3 ± 0.27	5 ± 0.27	4 ± 0.27

^a^Ciprofloxacin, Cephradine, Erythromycin and Polymyxin B were used as standard antibacterial agents at 20 µg/mL

^a^ND: not determined

As pure LLO is more efficient, it is essential to investigate its activity at different concentrations (2–10 U/mL) to estimate the MIC value. Evaluation of MIC value against each bacterial pathogen was implemented in comparison to the standard antibiotic agent (positive control) and without any treatment (negative control). As shown in Table 11, the significant ability of LLO to suppress the bacterial growth was conducted at lower MIC values. Since, a higher sensitivity of LLO was detected against *E. faecalis* (1.99 ± 0.85), *E. coli* (2.25 ± 0.22), *S. aureus* (3.12 ± 0.13), and *S. typhimurium* (5.33 ± 1.07), respectively. Accordingly, the LLO proved to be more active when the LLO dose increases. Since MIC values (1.99–5.33 U/mL) were 2–4 folds lower than that of ciprofloxacin reflected the strong antibacterial activity of the targeted enzyme.

**Table 11:** Minimum inhibitory concentration (MIC) of the potent synthesized derivatives

Sample No	Minimum Inhibitory Concentration (MIC)			
	***Staphylococcus aureus*** **MRSA**	***Enterococcus faecalis***	***Escherichia coli***	***Salmonella typhimurium***
Purified enzyme (U /mL)	3.12 ± 0.13	1.99 ± 0.85	2.25 ± 0.22	5.33 ± 1.07
Ciprofloxacin (µg/mL)	11.3 ± 3.44	8.8 ± 3.77	6.9 ± 0.14	4.5 ± 1.91

The purified LLO displayed superior catalytic efficiency, evidenced by a low Michaelis constant (***K***_***m***_) of 0.050 mM for its primary substrate, L-lysine. This affinity is notably higher than that reported for other microbial LLOs, such as the enzyme from *Trichoderma viride* (***K***_***m***_ = 0.12 mM), indicating a potentially stronger substrate-enzyme interaction in our marine bacterial variant. The comparative advantages of this *Streptomyces*-derived LLO are summarized in Table 11. While the potent antibacterial activity is observed (MIC 1.99–5.33 U/mL) (Table 12).

**Table 12:** Comparative biochemical and antimicrobial properties of L-lysine α-oxidase from various microbial sources

Enzyme Source	*K*_*m*_ (mM)	Optimal pH	Antibacterial MIC (U/mL)	References
Marine *Streptomyces griseobrunneus* (This Study)	0.05	6.4	1.99–5.33	This study
*Trichoderma viride*	0.12	7.5	~ 8.5	Kusakabe et al., 1980 [15]
*Trichoderma cf. aureoviride*	0.08	8.0	~ 2.0 *	Pokrovsky et al., 2013 [24]
*Trichoderma harzianum*	0.10	8.0	Study confirmed antibacterial activity but did not report a standard MIC value in U/mL	Senyagin et al., 2023 [5]
*Rhodococcus* sp. AIU Z-35–1	0.062	7.0	not reported for antibacterial activity	Isobe & Nagasawa, 2007 [10]
*Pseudomonas* sp. AIU 813	0.11	7.0	not reported for antibacterial activity	Isobe et al., 2012 [16]

## Conclusion

The marine isolate *Streptomyces griseobrunneus* S15 represents a robust source of novel LLLO with distinctive biochemical properties. The enzyme's high catalytic efficiency, evidenced by its low ***K***_***m***_ value and broad substrate range, surpasses many reported fungal LLOs. Its stability under physiological conditions and resistance to SDS suggest structural robustness for practical applications. The potent antibacterial activity against drug-resistant strains, particularly *Enterococcus faecalis* and *Escherichia coli*, highlights its therapeutic potential in an era of increasing antibiotic resistance. Unlike fungal LLOs with narrow specificity, this bacterial variant’s ability to oxidize multiple amino acids may enhance its efficacy in anticancer strategies by depleting tumor nutrients. This study has not evaluated the toxicity of LLO to mammalian cells, and systematic safety assessments are required to further explore its clinical application potential. Therefore, future research should look at its in vivo antibacterial efficacy using animal infection models and conducting comprehensive toxicity studies on mammalian cells to assess clinical safety. Direct measurement of H₂O₂ production during bacterial inhibition assays to conclusively establish this causal relationship and further elucidate the precise antimicrobial mechanism. Furthermore, applying enzyme immobilization strategies could enhance its stability and reusability, establishing a practical foundation for potential industrial or biomedical applications. This work expands the repertoire of microbial oxidases and provides a foundation for developing novel biocatalysts and antimicrobial agents.

## Data Availability

This article has all the data that was created or evaluated during this investigation.
